# A cluster randomised controlled trial and process evaluation of a training programme for mental health professionals to enhance user involvement in care planning in service users with severe mental health issues (EQUIP): study protocol for a randomised controlled trial

**DOI:** 10.1186/s13063-015-0896-6

**Published:** 2015-08-13

**Authors:** Peter Bower, Chris Roberts, Neil O’Leary, Patrick Callaghan, Penny Bee, Claire Fraser, Chris Gibbons, Nicola Olleveant, Anne Rogers, Linda Davies, Richard Drake, Caroline Sanders, Oonagh Meade, Andrew Grundy, Lauren Walker, Lindsey Cree, Kathryn Berzins, Helen Brooks, Susan Beatty, Patrick Cahoon, Anita Rolfe, Karina Lovell

**Affiliations:** NIHR School for Primary Care Research, Manchester Academic Health Science Centre, University of Manchester, Williamson Building, Oxford Road, Manchester, M13 9PL England; School of Nursing, Midwifery and Social Work, University of Manchester, Jean McFarlane Building, Oxford Road, Manchester, M13 9PL England; Centre for Biostatistics, Institute of Population Health, University of Manchester, Jean McFarlane Building, Oxford Road, Manchester, M13 9PL England; School of Health Sciences, University of Nottingham, Queen’s Medical Centre, Nottingham, NG7 2HA England; Faculty of Health Sciences, University of Southampton, Highfield, Southampton, SO17 1BJ England; Centre for Health Economics, Institute of Population Health, University of Manchester, Jean McFarlane Building, Oxford Road, Manchester, M13 9PL England; Manchester Mental Health and Social Care Trust, Chorlton House, 70 Manchester Road, Chorlton-cum-Hardy, Manchester, M21 9UN England

**Keywords:** Care planning, Severe mental illness, Service user involvement

## Abstract

**Background:**

Involving service users in planning their care is at the centre of policy initiatives to improve mental health care quality in England. Whilst users value care planning and want to be more involved in their own care, there is substantial empirical evidence that the majority of users are not fully involved in the care planning process. Our aim is to evaluate the effectiveness and cost-effectiveness of training for mental health professionals in improving user involvement with the care planning processes.

**Methods/Design:**

This is a cluster randomised controlled trial of community mental health teams in NHS Trusts in England allocated either to a training intervention to improve user and carer involvement in care planning or control (no training and care planning as usual).

We will evaluate the effectiveness of the training intervention using a mixed design, including a ‘cluster cohort’ sample, a ‘cluster cross-sectional’ sample and process evaluation. Service users will be recruited from the caseloads of care co-ordinators.

The primary outcome will be change in self-reported involvement in care planning as measured by the validated Health Care Climate Questionnaire. Secondary outcomes include involvement in care planning, satisfaction with services, medication side-effects, recovery and hope, mental health symptoms, alliance/engagement, well-being and quality of life. Cost- effectiveness will also be measured. A process evaluation informed by implementation theory will be undertaken to assess the extent to which the training was implemented and to gauge sustainability beyond the time-frame of the trial.

**Discussion:**

It is hoped that the trial will generate data to inform mental health care policy and practice on care planning.

**Trial Registration Number:**

ISRCTN16488358 (14 May 2014)

## Background

Involving service users in their own care and allowing choice is at the centre of policy initiatives aimed at improving quality of care and enhancing recovery. This principle is enshrined and prioritised in health care [1, 2], particularly mental health care policy and guidelines [3, 4]. Current guidance states that the outcome of any assessment will be a care plan developed with the service user, the professional/member of the care team and other appropriate parties, such as families and carers [5, 6]. Mental health nurses, psychiatrists and allied health and social care workers provide the majority of care for service users regardless of setting. They have a pivotal role in the development of care planning, particularly as care co-ordinators under the Care Programme Approach (CPA) [5, 6]. Despite the consensus among policy-makers, professionals, service users and carers about the importance of involvement there is substantial empirical evidence that the majority of users and carers are marginalised in the care planning process. This lack of involvement occurs in both in-patient and community settings [7, 8] and there is evidence that current models of involving users and carers in their care are less effective than first envisaged.

One of the key findings of the Care Quality Commission’s national survey, which included 7500 users of mental health services, was that service users were often not involved in their care as much as they wanted to be, with only 34 % agreeing that they were ‘definitely’ involved as much as they wanted to be in decisions about their care and treatment [7, 8]. A significant number of studies of user and carer experience echo the need and desire to be involved in their care [9], but despite this evidence there has been little change in practice. There is also evidence that carers feel excluded, unsupported and distanced by mental health services and want to be more involved in the care planning process [10–13]. A systematic review of user and carer views and expectations of mental health nurses, including 132 papers over a 20-year period [14], found that users and carers expressed a need for openness and adequate information, more choice and increased involvement in the assessment and planning of their care. Perhaps most importantly it found that the literature remains strikingly consistent over time, indicating a sustained lack of impact on practice.

This research aims to develop a standardised training intervention (EQUIP training) to improve user and carer involvement with the care planning process, and to test the effectiveness and cost-effectiveness of this package. The study is a pragmatic effectiveness and cost-effectiveness trial and, thus, the optimal comparator is a no training control representing usual practice.

If proven effective and cost-effective, it will provide a model for wider implementation to improve the quality of mental health care across community mental health services.

### Objectives

To conduct a cluster randomised controlled trial (RCT) of effectiveness and cost-effectiveness of the EQUIP training for improving involvement of service users and carers compared with controls in UK National Health Service (NHS) community mental health services.

## Methods/Design

**Fig. 1:**
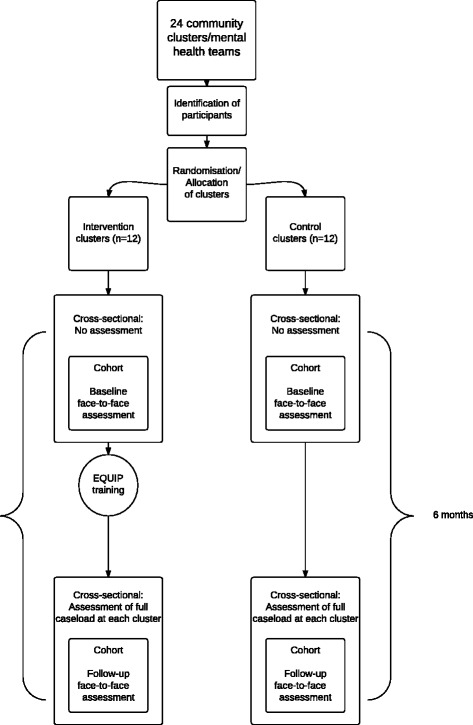
EQUIP trial design

**Fig. 2:**
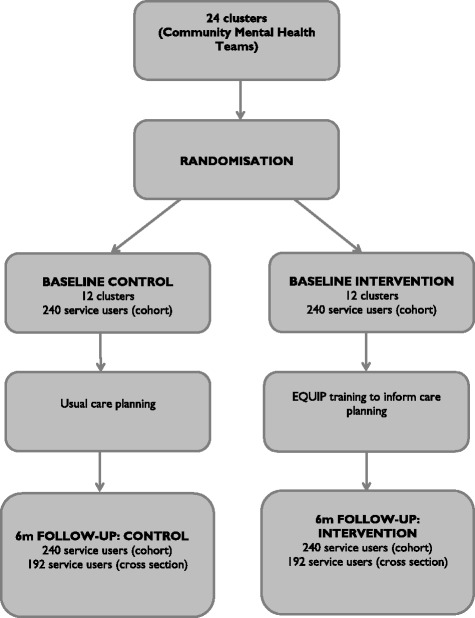
Consolidated Standards of Reporting Trials (CONSORT) diagram

As the trial involves a health professional training intervention, a cluster randomised design is required to avoid contamination. A mixed design, including a ‘cluster cohort’ design, a ‘cluster cross-sectional’ design and process evaluation will be adopted (see Fig. 1 for an outline of the trial design, and Fig. 2 for the Consolidated Standards of Reporting Trials (CONSORT) flow diagram). Adoption of the combined design provides protection against problems in either of the individual approaches.

In the ‘cluster cohort’ design, service users cared for by each Community Mental Health Team (CMHT) will be recruited, and a detailed face-to-face assessment will be conducted at baseline. Carers will be recruited from consenting service users. Each CMHT will then be randomised to either intervention (training in care planning) or control (usual care planning). The CMHTs randomised to intervention will then receive the EQUIP training. Six-month follow-up face-to-face assessments will then be conducted with the same service users and carers (intervention and control groups) 6 months after the baseline assessment.

For the cluster cohort, service users need to participate in two face-to-face assessments, which may be burdensome to service users. This means the cluster cohort may be vulnerable to recruitment difficulties (i.e., only a small number of eligible service users take part) and attrition (i.e*.*, service users do not attend for follow-up), reducing both sample size and external validity.

The design will, therefore, also include a ‘cluster cross-sectional’ element to help ameliorate problems of recruitment and attrition within the cluster cohort. Six months after randomisation, a smaller number of questionnaires (see Table 1) will be posted to all service users who are not part of the ‘cluster cohort’ but who are under the care of all participating teams.

**Table 1:** Outcome measures used with the 3 sample groups at baseline and 6-month follow-up

Sample	Baseline	Six months
Service user cohort (face-to-face collection)	Demographic data	
	Primary outcome:	Primary outcome:
	HCCQ-10	HCCQ-10
	Secondary outcomes:	Secondary outcomes:
	VSSS-EU-54	VSSS-EU-54
	GASS	GASS
	WEMWBS	WEMWBS
	DREEM	DREEM
	HADS	HADS
	CALPAS-12	CALPAS-12
	WHOQOL-BREF	WHOQOL-BREF
	PROM	PROM
	Economic outcome:	Economic outcome:
	EQ-5D-5L	EQ-5D-5L
	Service use questionnaire	Service use questionnaire
Carer cohort (postal collection)	Demographic data	
	Secondary outcomes:	Secondary outcomes:
	PROM	PROM
	CUES-C	CUES-C
	WHOQOL-BREF	WHOQOL-BREF
	Economic outcome:	Economic outcome:
	EQ-5D-5L	EQ-5D-5L
Cross-sectional sample: service users (postal collection)		Demographic data
		Primary outcome:
		HCCQ-10
		Secondary outcome:
		PROM (14-item SF version)
		Economic outcome:
		EQ-5D-5L
		Service use questionnaire

*CALPAS-12* California Psychotherapy Alliance Scales, *CUES-C* Carers’ and Users’ Expectations of Services – carer version, *DREEM* Developing Recovery Enhancing Environments Measure, *EQ-5D-5L* EuroQol-5D-5L, *GASS* Glasgow Antipsychotic Side-effect Scale, *HADS* Hospital Anxiety and Depression Scale, *HCCQ-10* Health Care Climate Questionnaire, *PROM* Patient Reported Outcome Measure, *WHOQOL-BREF* World Health Organisation Quality of Life*.*, *VSSS-EU-54* Verona Service Satisfaction Scale, *WEMWBS* World Health Organisation Quality of Life

Service users in the cross-sectional sample only have to agree to assessment once, and the assessment is designed to be less burdensome as it includes fewer measures. This means that a higher proportion of service users may agree to take part in the study, potentially giving a larger sample size.

However, the ‘cluster cohort’ design allows adjustment for a wider range of baseline characteristics at an individual level because of the greater range of measures included, giving potentially greater statistical power compared to analysis of the ‘cluster cross-sectional’ data [15]. Comparative analyses of data from the two designs will allow the possibility of pooling results from the two designs to be investigated.

A potential threat to the validity of a cluster randomised trial is recruitment bias, where professionals allocated to different trial arms recruit differently depending on their allocation, leading to selection bias and baseline incomparability [16]. Whilst it is preferable to recruit service users prior to allocation, the logistics of the trial means that clusters will need advanced notice of their training date, which will require us to inform them of their allocation. Therefore, initial patient selection in the EQUIP trial is not by those being trained in the trial, but will use existing registers of service users. This will be undertaken by NIHR Clinical Research Network Clinical Studies Officers (CSO) and Trust staff. This will limit the opportunity for biased recruitment. Carers will be recruited by providing service users with a recruitment pack to give to their carer (if a carer is identified and wishes to consider taking part).

A longitudinal process evaluation of the training programme is being undertaken which incorporates multiple interviews over time, observations and diary methods of data collection. This will run alongside the trial and is being conducted because successful implementation of user and carer-led care planning implicates a range of factors including the integrity of the intervention and the acceptability of the intervention to both clinicians and service users [17].

### Study setting and inclusion criteria

#### Community Mental Health Teams (CMHTs)

The study is being conducted in NHS Trusts in the UK.

All CMHTs within the participating NHS Trusts will be eligible for inclusion. Service users aged 18 and over with a severe mental illness (e.g., psychosis, bipolar disorder, schizophrenia) under the care of participating CMHTs will be eligible for inclusion. We will seek consent from service users to access health records to collect data on diagnosis, service use and treatment history.

### Intervention design – User/carer-led training package to inform care planning

All consenting CMHTs allocated to the intervention arm will receive the EQUIP training intervention developed in our earlier work. The EQUIP training intervention consists of 2 days face-to-face training, an 8-hour optional self-directed learning package and 6 hours supervision per team in the 6 months following the training. In recruitment of teams, we ask that at least 80 % of staff designated as ‘care co-ordinators’ (i.e., those with a caseload) commit to attend the training. We will document attendance at training by all professionals.

### Comparator – Usual care

This will consist of ‘usual practice’ in care planning, without access to the specialist training described above. We will have considerable detail about what ‘usual practice’ consists of and how it varies from unit to unit from the embedded process evaluation.

### Intervention components

We describe the intervention according to the Template for Intervention Description and Replication (TIDieR) guidelines [18]:

- Why: our aim was to co-develop, co-produce and co-deliver (with service users/carers) a best evidence, acceptable and feasible training programme for mental health professionals to enhance user and carer involvement in care planning. Two reviews were conducted including: a narrative synthesis (Bee et al., in press) which examined how user-involved care planning is operationalised within mental health services and to establish where, how and why challenges to user involvement occur; and a scoping review of training reviews and interventions that change clinician behaviour. In addition, focus groups and individual interviews with service users, carers and health professionals were conducted to ascertain training content and delivery requirements and to determine the priorities and components of adequate user and carer involvement in care planning. The evidence from the reviews and qualitative data was synthesised to develop and design the training.
- What: a range of training materials have been developed for the training including PowerPoint slides, case scenarios, audio-recordings from health professionals, service users and carers and a trainer’s manual.
- Who: the synthesis identified that the training should be multi-disciplinary, including all health professionals and psychiatrists. Team training was seen as optimal and, as far as possible, teams will be trained together. The training will be delivered by 2 of the co-applicants (both academics with teaching experience) and 3/4 service users and carers who have attended a 4-day ‘train the trainers’ course.
- How: the synthesis indicated that training should include a range of formats: face-to-face, self-directed learning and follow-up supervision. The consensus exercise indicated a minimum of 15 hours and maximum of 30. The course will run for 2 days (12 hours) plus 6 hours follow-up supervision and 8 hours self-directed learning (optional). Hence, each health professional will receive 18 hours of facilitated training and an additional optional 8 hours self-directed learning.
- Where: consensus was reached that the training venue should be outside the clinical area, geographically convenient, provide good catering and in a venue with appropriate training resources.
- When and how: the training will be delivered to each cluster randomised to the training intervention over 2 days. In recruiting teams, we ask that 80 % of the care co-ordinators within each team attend the training. The training will be delivered within 6 weeks of service users being recruited into the trial.
- Tailoring: the intervention has been tailored for health professionals.
- Modifications: only minor modifications will be made in light of feedback during the trial. If the trial is successful and we implement the training across other NHS Trusts, modifications will be made in light of feedback collected from the process evaluation.
- How well: fidelity of the training has been ensured by the careful development and synthesis work described earlier, the ‘train the trainers’ course, the development of a detailed manual and the delivery of training by the same groups of trainers.

### Outcome assessment

#### Primary outcome

The Health Care Climate Questionnaire (HCCQ-10) [19] is the primary outcome measure for the service users in the trial.

The HCCQ-10 was developed to assess patient experience of health care and the degree to which their care offers support for autonomy. The scale has ten items, which are scored on a seven-point scale ranging from ‘strongly disagree’ to ‘strongly agree’. An overall score is calculated as the mean of the items (expressed out of 100), where a higher score indicates greater support for autonomy.

#### Secondary outcomes

Secondary outcome measures were determined using experts and a consensus discussion exercise with the service user/carer advisory group. Key domains to measure were recommended by the service user/carer advisory group in our earlier Programme Development Grant (RP-DG-1209-10020). The domains identified were: quality of life; alliance/engagement; service satisfaction; well-being; mental health symptoms; hope and recovery; and medication side-effects. Six of these domains have one questionnaire selected for completion, whilst the domain ‘satisfaction’ has separate questionnaires for both service users and carers.

A new measure of user involvement in care planning, the Patient Reported Outcome Measure (PROM) was developed in consultation with our user and carer advisory group. The need for this measure was determined during the development of this grant as existing measures of user involvement were not deemed adequate by the advisory group as they failed to sufficiently incorporate the views of users. The newly developed PROM will be included as a secondary outcome to measure user and carer involvement in care planning. The new measure has been validated using the Rasch model and has displayed excellent psychometric and scaling properties [20]. The 61-item scale (and 14-item short form version) is suitable for both service users and carers. Items are scored on a five-point Likert scale from ‘Completely disagree’ to ‘Completely agree’. Higher scores reflect greater service user and carer involvement with care planning. Data from this study will provide further evidence of the acceptability, validity and sensitivity to change of this measure for this population.

#### Satisfaction (service users)

Verona Service Satisfaction Scale (VSSS-EU-54) [21, 22], is a validated, multi-dimensional, self-administered scale for measuring service users’ satisfaction with mental health services. There are seven dimensions: overall satisfaction, professional skill and behaviour, access, efficacy, types of intervention and relatives’ involvement. Participants are asked to express their overall feeling about their experience of the mental health service they have been attending in the last year. Satisfaction ratings are on a five-point Likert scale, with higher scores representing greater satisfaction. Global and subscale scores can be obtained. Reliability testing has shown that the VSSS-EU-54 has good internal consistency and stability.

#### Satisfaction (carers)

Carers and Users’ Expectations of Services - carer version (CUES-C) [23] will be used to measure carers’ views of services. There are three parts to the questionnaire; part A measures the impact of caring, part B measures the quality of support provided by carers and part C is a free text response for advice and help. The self-rating scales consist of 13 items each in parts A and B, totalling 26 questions. All questions are answered using a three-point scale. Scores for each part range from 0 to 26, with higher scores representing more dissatisfaction and the need for more support. The scale has been found to be suitable to use to assess carers’ experiences.

#### Medication side-effects

The Glasgow Antipsychotic Side-effect Scale (GASS) [24] is a self-rating scale to detect the side-effects of antipsychotic medication. The scale consists of 22 questions and scores range from 0 to 66. Higher scores reflect more frequent experience of side-effects, with total scores providing three categories of severity (absent/mild side-effects, moderate side-effects and severe side-effects).

#### Well-being

The Warwick-Edinburgh Mental Well-being Scale (WEMWBS) [25] is a short, psychometrically robust scale, which is easy to complete. It has 14 items scored on a 5-point Likert scale ranging from ‘none of the time’ to ‘all of the time’ based on experience over the past 2 weeks. Scores range from 14–70 and a higher score indicates a higher level of mental well-being.

#### Recovery and hope

Developing Recovery Enhancing Environments Measure (DREEM) [26] is a self-report measure used to assess mental health recovery of people who receive mental health services. It is a 166-item questionnaire which is organised into 24 subscales (such as ‘stage of recovery’ and ‘elements of recovery’), including a final section consisting of open-ended questions. Responses are scored on a five-point Likert scale ranging from ‘strongly agree’ to ‘strongly disagree’, with low scores representing more positive experience.

#### Symptoms

Hospital Anxiety and Depression Scale (HADS) [27] is a 14-item scale using a 4-point Likert scale. Items are added to give two scores, one for anxiety and one for depression, with higher scores representing more severe symptoms. Scores range from 0 to 21 for both anxiety and depression. This is a well-used and validated measure.

#### Alliance/engagement

California Psychotherapy Alliance Scale (CALPAS) [28] is a 12-item, self-report questionnaire, which provides a total score. It has four subscales: ‘service user capacity to work purposefully in therapy’, ‘the affective bond with the therapist’, ‘therapist’s empathic understanding’ and ‘involvement and the agreement between the patient and therapist on the goals and tasks of treatment’. Each item is rated on a 6-point Likert scale, with scores ranging from 12 to 84, with higher scores representing better alliance. It has good reliability and validity.

#### Quality of life

World Health Organisation Quality of Life (WHOQOL-BREF) [29] is a 26-item questionnaire consisting of 4 domains (physical, psychological, social relationships and environment). Each question uses a 5-point Likert scale, ranging from a score of 1 to 5, with higher scores representing more positive ratings. Total scores are computed within each domain. It has been shown to demonstrate good reliability and validity.

### Economic outcome

#### Health Status

The EQ-5D-5L [30] will be used to assess health status and to estimate Quality-Adjusted Life Years (QALYs), using published EQ-5D utility weights for the UK. QALYs will be the primary measure of health benefit for the economic analysis. The EQ-5D-5L, has two parts; part 1, a 5-item questionnaire consisting of 5 dimensions (mobility, self-care, usual activities, pain/discomfort and anxiety/depression). Each dimension has five levels, ranging from no problems to severe problems. The five dimensions can be combined to describe the respondents’ health state. Part 2 is a visual analogue scale (VAS) which records respondents’ self-rated health on a vertical VAS, where the end points are labelled, ‘best imaginable health state’ and ‘worst imaginable health state’.

#### Service use questionnaire

A Service Use Questionnaire will be used at baseline and follow-up to identify the range of services used by each trial participant and how much they have used each service. The measure will be used to estimate the costs of health and social care service use.

### Use of outcome measures and methods of data collection

Use of outcome measures with the three sample groups (service user cohort, carer cohort, cross-sectional cohort) is further detailed in Table 1.

#### Service user cohort sample

Demographic data, primary, secondary and economic outcome measures will be collected via a face-to-face assessment. As the ‘cluster cohort’ assessment is more burdensome, service users will receive a £10 voucher for their time after completion of the follow-up interview at 6 months.

#### Carer cohort sample

Demographic data, primary, secondary and economic outcome measures will be collected via a postal method. Carers will receive a £5 voucher following receipt of the follow-up questionnaires at the 6-month time point.

#### Cross-sectional sample (service users)

Demographic data, primary, secondary and economic outcome measures will be collected via a postal method at the 6-month time point. There will be no collection of carer information in the cross-sectional part of the study. Respondents can choose to receive a £5 voucher on receipt of the questionnaire by providing postal contact details.

Table 1 indicates the measures to be used, with which respondents, and at what time points.

### Sample size

The primary outcome is the Health Care Climate Questionnaire (HCCQ-10), identified by our user consultation group as their preferred outcome measure. However, data on the use of this scale with service users with severe mental illness is limited and so we have used a standardised effect to calculate sample size and power. A trial with 12 clusters per arm and a mean of 20 service users per cluster (total sample size of 480) will provide power greater than 80 % to detect a standardised effect size of 0.4. This assumes an intra-cluster correlation coefficient of 0.05 and an 80 % follow-up rate, giving 384 participants with complete data in the analysis. Power will be increased by inclusion of baseline covariates (see Statistical analysis below).

For the cross-sectional component, the primary outcome remains the HCCQ-10. We aim to recruit at least the same number of participants in each cluster; given that the same clusters will be assessed as the cohort component. As with the cohort component a sample size of 384 in the cross-sectional component will ensure power greater than 80 % in the corresponding analysis. Any additional data gathered should increase power.

### Recruitment

Trust managers have agreed that we can recruit CMHTs. To recruit professionals we will use our applicants to champion the study. Teams will be introduced to the trial via a letter of support from the chief executive, and/or via meetings with senior managers. Meetings will also be held with area team managers (and if requested, staff) across both sites to facilitate engagement with and understanding of the trial.

Recruitment and training will be undertaken in sequence, to maximise efficiency in delivery of training to community teams, but also to ensure there is sufficient time to permit the relevant baseline assessments to be undertaken with service users (see below). CMHTs will be randomly allocated to receive the EQUIP training or control.

#### Recruitment of service users and carers - Cluster cohort

Service users will be excluded if their participation is judged as inappropriate by the CMHTs: for example, if a patient is not deemed to have capacity to provide fully informed consent or is too unwell at the time of recruitment. We will seek to document all exclusions and report them as part of the trial CONSORT diagram.

Any carer (aged 18 years or older) of the service user will be eligible for inclusion in the ‘cluster cohort’ study if they agree to take part. Consent will be implied by response via completion and return of the baseline questionnaires (and return of the consent to contact form if they wish to be contacted at 6-month follow-up).

To recruit service users in the ‘cluster cohort’, the direct care team within the CMHTs (cluster) will produce a list of all service users who meet eligibility criteria to participate in the trial, including any reasons for exclusions. This list will be referred to as their ‘caseload’. These patient lists will be used by the NIHR Clinical Research Network CSOs to send out an introductory letter, participant information sheet and consent to contact form to each patient, inviting them to take part in the study. Service users will be required to ‘opt in’ by returning the consent to contact form in a pre-paid envelope to the research team. CSOs will contact non-responders by telephone on one occasion to additionally allow service users to opt in over the telephone. The research team will then follow-up the consent to contact forms, to answer any further questions, and when a participant is recruited, the researcher will continue with a face-to-face informed consent process. Following signed consent, service users will be invited to complete all baseline measures.

Service users will be asked at the informed consent meeting to nominate a carer to be included in the study and, if they choose to do so, will be provided with a questionnaire pack (including introductory letter, information sheet, questionnaire, pre-paid envelope and consent to contact at 6-month follow-up form).

Service users who consent to take part in the trial will be offered the opportunity to take part in the process evaluation based on a purposive sampling framework to ensure variation in gender, age and randomised group. Potential participants will be provided with an invitation letter, information sheet and consent to contact form during the baseline meeting.

#### Recruitment of service users – Cluster cross-sectional

To recruit service users in the ‘cluster cross-sectional’ study, we will conduct a postal survey of all service users under the care of each community mental health team 6 months after randomisation, excluding those already recruited to the ‘cluster cohort’.

### Sequence generation and allocation concealment

Once service users and carers have been sent a trial invitation pack (following caseload review), the clusters will be allocated randomly to either intervention or control. To eliminate selection bias, allocation will be determined through an external telephone randomisation service at the Clinical Trials Unit of the Manchester Academic Health Science Centre.

Clusters will be submitted to the randomisation service in pairs. Each pair will be from the same site and similarly matched in other characteristics where possible, to reduce imbalance between intervention and control arms. One member of the pair will be allocated to intervention by random selection, the other allocated to control.

### Blinding

To reduce detection bias, we will seek to blind researchers undertaking assessments of the quality of care planning to the allocation. We will report the success or otherwise of our attempts at blinding.

### Data management

All data will be stored securely in line with local data management arrangements. All questionnaires and other paper records will be stored in secure storage facilities at the University of Manchester and the University of Nottingham. Personal identifiable paper records will be stored separate from anonymised paper records. All electronic records will be pseudo-anonymised using a reference number for each participant and stored on a password protected server at the University of Manchester.

#### Statistical analysis

Analysis of outcomes will follow intention-to-treat principles: outcome data will be sought and included in the analysis for all service users irrespective of receipt of the intervention or completion of care planning during the time scale of the EQUIP trial.

Standard data checking procedures will be used as part of the data cleaning procedure prior to locking the database and linkage to group allocation. We will then model the pattern of missing data in terms of baseline characteristics of service users and treatment allocation to check for differential non-response. Depending on the patterns of missing data we may at this point choose to use multiple-imputation. Multiple-imputation may also be used in sensitivity analyses for non-ignorable missing data assumptions [31].

For the cluster cohort study the intervention effects for the primary outcome (HCCQ-10) and secondary outcome measures will be estimated using a linear mixed model with a random intercept for CMHTs. The baseline value of the outcome will be used as a covariate together with other covariates pre-specified in the statistical analysis plan. The same statistical modelling procedure will be used for estimation of the intervention effect in the cluster cross-sectional study using a restricted set of covariates. Full detail of covariates for each model will be confirmed in the statistical analysis plan. We will then test for heterogeneity of the treatment effect using the combined cohort and cross-sectional data. If there is no evidence of heterogeneity, we will estimate a pooled treatment effect.

A draft statistical analysis plan for primary and secondary outcomes, including sub-group analyses will be presented to and agreed with the Programme Steering Group prior to the commencement of the data analysis.

### Economic analysis

A cost-effectiveness acceptability analysis will be conducted from the perspectives of health and social care providers and service users, the key stakeholders in treatment decisions. The time horizon for the primary economic analysis will be at scheduled follow-up (6 months). Data on service use and health status (EQ-5D-5L) for the economic analysis will be collected for all participants at baseline and follow-up and via the cross-sectional survey. The economic analysis will control for key covariates that are associated with either costs or QALYs. Bootstrap techniques will be used to estimate the cost-effectiveness acceptability curve, likelihood that the intervention could be cost-effective and net benefit statistic.

The objective of the economic study will be to assess the relative cost-effectiveness of the training intervention to improve patient outcomes and/or reduce the costs of health and social care.

### Process evaluation

A process evaluation of the training programme delivered as part of the trial is deemed appropriate because successful implementation of the user/carer-led care planning training implicates a range of factors including the integrity of the intervention and the acceptability of the intervention to both clinicians and service users [17].

The process evaluation is designed to explore by how far the user/carer-led care planning has been taken up and implemented in the daily work of the health professionals who attended the training and what the consequences of this uptake have been. It will complement and supplement the evidence provided by the main randomised trial, as recommended by the Medical Research Council (MRC) framework for evaluation of complex intervention [32]. The design and execution of the process evaluation will draw on Normalisation Process Theory (NPT) [33] from the outset as well as other theories of implementation science. NPT comprises four components: coherence (sense making work), cognitive participation (relational work), collective action (operational work), reflexive monitoring (appraisal work). NPT has been developed from empirical studies of the implementation of complex interventions in health care contexts and in relation to mental health contexts in particular. We will focus on: (a) implementation of user/carer-involved care planning – the way this is developed and translated into practices (of mental health professionals, users, carers and others); (b) embedding – the manner in which care planning becomes, (or does not become), routinely incorporated in everyday work of service users and professionals; (c) integration – how care planning is sustained as part of the everyday lives of individuals at work and at home; and d) networking – how it generates access to new networks and resources.

### Informed consent

The key ethical concerns for the programme include confidentiality, participant anonymity and informed consent to participate. The study includes both mental health service users and carers as participants and, as such, there are specific ethical issues to be considered. Research governance principles and ethical committee approvals bind all applicants and institutions. We will ensure we adopt the highest standards of research conduct including involvement of service user representation in the management and delivery of the research.

The study will be conducted in compliance with the study protocol, Good Clinical Practice (GCP) and both University and NHS regulatory and monitoring requirements. The Work stream teams will meet every 3 months and the chief investigator (CI) will be responsible for the overall leadership, management and outputs of the programme. The principal investigator (PI) from each site will maintain a log of the key milestones to be achieved against the timetable. The programme managers and Work stream leads will be responsible for the day to day running and co-ordination of the studies and will be accountable to the PI. All research associates will be supervised by the Work stream leads.

All potential participants will be provided with an information sheet written to current NRES guidelines and favourably reviewed by the relevant ethics committee, prior to the study commencing. Service users and carers have been involved in developing the participant information sheets to ensure they are accessible. The information sheet will be provided to potential participants at the point of them expressing an interest in participating. It will provide potential participants with information about the study, including the potential benefits and risks of taking part, confidentiality and the right to withdraw. Researcher contact details will be provided so participants can contact them with any queries prior to the participant deciding whether or not to take part. Researchers will further discuss risks and benefits immediately prior to the data collection taking place.

Participants in randomised trials usually provide written informed consent for a range of research procedures, including participation in the trial, randomisation and data collection. However, conventional informed consent procedures are not always appropriate in the context of a cluster, randomised trial [34]. In the EQUIP trial, community mental health services are making the decision to take part in the EQUIP trial and agree to randomised allocation. This is described as a ‘cluster cluster’ design, and is distinguished from an ‘individual cluster’ design [35]. In the latter, randomisation is at the level of the cluster, but specific services are delivered to individuals, and service users can consent to receive or not receive that intervention. The recent CADET trial was an example of an individual cluster design [36]. In ‘cluster cluster’ designs such as the EQUIP trial, service users cannot opt out of a cluster in the same way, as the community teams will have been trained in the new methods. The recent WISE study was an example of this design [37].

In the EQUIP trial, seeking formal consent for participation and randomisation may, therefore, be inappropriate, as these processes are not under control of the service users. The following consent procedures will be adopted.

If the service, and the CMHTs consent to take part in the trial, then individual service users will not be asked for specific consent to be randomised as part of the EQUIP trial. Service users cannot, therefore, ‘opt out’ of their cluster allocation. The Clinical Research Network CSOs (or Trust staff) will be responsible for accessing patient details and determining who is eligible to take part in the study and be contacted. They will be responsible for sending out information about the study to the identified service users, along with an invitation to participate. The research team will not have access to service user details until they have returned the consent to contact form.

Service users in the ‘cluster cohort’ will undertake a detailed face-to-face assessment at 2 points in time (baseline and 6 months). For these, a formal written consent procedure will be adopted. It will be explained to service users that their CMHT is involved in a study to test the effectiveness of a new training package on service user/carer involvement in care planning compared to the usual care planning experience. Participants will be told that we will do this by delivering the training to some mental health teams and not to other teams to see if receiving the training has an impact on the extent of service user/carer involvement in care planning.

Carers in the ‘cluster cohort’ will undertake a postal survey at baseline and at 6 months. Carers will be asked to participate via nomination by a service user. Response to the questionnaires will be treated as consent. Carers will also be asked to complete a consent to contact form to allow invitation to complete the follow-up questionnaire at 6 months, which is returnable to the research team in the prepaid envelope provided.

Service users in the ‘cross-sectional sample’ will undertake a short postal survey at 6 months only. For these, we will treat this part of the study as a survey. Service users will receive a postal invitation to the survey, seeking their views on the quality of the care planning process that they have received. Response to the survey will be treated as consent, as is usual in survey work. Participants may change their mind and withdraw from the study at any point and this will not affect the care they receive.

### Ethics

The study received a favourable ethical opinion from the National Research Ethics Service (NRES) on 8 August 2014 (NRES Committee North West Lancaster, REC Reference 14/NW/0297; IRAS Project ID 125899). It will be conducted in accordance with the UK Department of Health Research Governance Framework in Health and Social Care (2005) and adhere to the ethical principles of the Helsinki Declaration (2013). All research staff involved in the conduct of the trial will meet the standards laid out in the ICH Tripartite Guideline for Good Clinical Practice (1996).

All data will be anonymised and secured off site following data input for a period of at least 5 years in accordance with the Data Protection Act (1995).

The design of this trial will not disadvantage service users in either the care planning training groups or control groups. No treatment is being withheld from service users in the control group.

### Ancillary studies

Data from an embedded sub-study will contribute to a programme of research (www.population-health.manchester.ac.uk/mrcstart/) funded by the MRC to expand the relatively small evidence base on an important issue concerning the recruitment of participants to trials [38].

It is recognised that patient and public involvement (PPI) is an important part of designing studies that are sensitive to the needs and preferences of patients, and the EQUIP study has invested significant time and resources into ensuring that PPI on the project receives sufficient focus. The embedded sub-study (‘START in EQUIP’, funded through an NIHR Doctoral Research Fellowship – award number DRF-2012-05-128) aims to evaluate whether telling eligible patients directly about the PPI in EQUIP increases patient recruitment into the study.

Service users will be randomised to receive a flyer detailing the extensive PPI in EQUIP alongside the standard participant information sheet, or the standard participant information sheet alone. This will be implemented by using cluster randomisation, in a crossed-factorial design with the main EQUIP allocation. We will test whether receiving the flyer is associated with higher levels of recruitment into EQUIP.

## Discussion

Involving service users in planning their care is at the centre of policy initiatives in the United Kingdom to improve quality of mental health care. Users value care planning and involvement in care, but achieving this in practice is far from straightforward.

Our aim is to evaluate the effectiveness and cost-effectiveness of training for mental health professionals in improving user involvement with the care planning processes. We have designed and delivered a pragmatic trial which will test this training and its impact in the context of routine care delivery. We have adopted very simple inclusion criteria to include as broad a range of patients as possible and enhance the pragmatic nature of the trial.

Conducting trials such as this in the context of routine care raises significant logistical challenges. Recruiting adequate numbers of patients in the limited recruitment window prior to training is proving challenging, and busy services can struggle to provide time for staff to attend the relevant training sessions when faced with significant clinical pressures.

The planned process evaluation will allow us to explore the impact of these pressures on delivery and implementation of the training. If successful in improving outcomes, we will be active in disseminating and implementing the results of the trial.

## Trial status

CMHTs in the first 18 clusters have been randomised and training of staff in the experimental arm of these clusters completed. Randomisation and training of the remaining clusters will continue until December 2015. Recruitment and data collection of primary and secondary outcomes with service users and carers began in July 2014 and will continue until June 2016.

## Abbreviations

CALPAS: California Psychotherapy Alliance Scales
CI: chief investigator
CMHT: Community Mental Health Team
CONSORT: Consolidated Standards of Reporting Trials
CPA: Care Programme Approach
CSO: Clinical Studies Officers
CUES-C: Carers’ and Users’ Expectations of Services - carer version
DREEM: Developing Recovery Enhancing Environments Measure
EQUIP: Enhancing the Quality of User Involved Care Planning in Mental Health Services
GASS: Glasgow Antipsychotic Side-effect Scale
GCP: Good Clinical Practice
HADS: Hospital Anxiety and Depression Scale
HCCQ-10: Health Care Climate Questionnaire
ICH: International Conference on Harmonisation
ISRCTN: International Standard Randomised Controlled Trial Number
MMHSCT: Manchester Mental Health and Social Care Trust
MRC: Medical Research Council
NHS: National Health Service
NIHR: National Institute for Health Research
NPT: Normalisation Process Theory
NRES: National Research Ethics Service
PI: principal investigator
PMG: Programme Management Group
PPI: Patient and Public Involvement
PROM: Patient Reported Outcome Measure
PSG: Programme Steering Group
QALYs: Quality-Adjusted Life Years
RCT: randomised controlled trial
REC: Research Ethics Committee
TIDieR: Template for Intervention Description and Replication
VAS: visual analogue scale
UK: United Kingdom
VSSS: Verona Service Satisfaction Scale
WEMWBS: Warwick-Edinburgh Mental Well-being Scale
WHOQOL: World Health Organisation Quality of Life
